# Regulation of cGAS/STING signaling and corresponding immune escape strategies of viruses

**DOI:** 10.3389/fcimb.2022.954581

**Published:** 2022-09-14

**Authors:** Zhe Ge, Shuzhe Ding

**Affiliations:** ^1^ School of Sport, Shenzhen University, Shenzhen, China; ^2^ Key Laboratory of Adolescent Health Assessment and Exercise Intervention of Ministry of Education, East China Normal University, Shanghai, China

**Keywords:** cGAS/STING signaling, innate immunity, virus, immune escape, PRRs

## Abstract

Innate immunity is the first line of defense against invading external pathogens, and pattern recognition receptors (PRRs) are the key receptors that mediate the innate immune response. Nowadays, there are various PRRs in cells that can activate the innate immune response by recognizing pathogen-related molecular patterns (PAMPs). The DNA sensor cGAS, which belongs to the PRRs, plays a crucial role in innate immunity. cGAS detects both foreign and host DNA and generates a second-messenger cGAMP to mediate stimulator of interferon gene (STING)-dependent antiviral responses, thereby exerting an antiviral immune response. However, the process of cGAS/STING signaling is regulated by a wide range of factors. Multiple studies have shown that viruses directly target signal transduction proteins in the cGAS/STING signaling through viral surface proteins to impede innate immunity. It is noteworthy that the virus utilizes these cGAS/STING signaling regulators to evade immune surveillance. Thus, this paper mainly summarized the regulatory mechanism of the cGAS/STING signaling pathway and the immune escape mechanism of the corresponding virus, intending to provide targeted immunotherapy ideas for dealing with specific viral infections in the future.

## 1 Introduction

Human beings have been fighting fiercely with viruses since ancient times. Today’s severe acute respiratory syndrome coronavirus 2 (SARS-CoV-2) seriously threatens human health and public safety. Innate immunity is the host’s first line of defense against pathogen invasion. Pattern recognition receptors (PRRs) are essential for mediating innate immune defense. The PRRs in the cell, including toll-like receptors (TLRs), nod-like receptors (NLRs), retinoic acid-inducible gene-I-like receptors (RLRs), C-type lectin receptors (CLRs), and DNA sensors, recognize the corresponding pathogen-related molecular pattern (PAMPs) to activate the relevant signal pathway to trigger type I Interferons (IFN-I), pro-inflammatory cytokines, and chemokines to eliminate invading pathogens (Carty et al., 2021). PAMPs are relatively conserved molecules in the evolution of pathogens. The main PAMPs are the nucleic acids of pathogens, including DNA (unmethylated CpG sequences), double-stranded RNA, single-stranded RNA, 5-triphosphate RNA (5’-ppp RNA), as well as lipoproteins, cell surface glycoproteins, membrane components, and viral capsids (Tang et al., 2012; Yoh et al., 2022). DNA is one of the vital genetic materials of the virus, which can release its DNA into the cytoplasm once it has infected the host cell. The cyclic GMP-AMP synthase (cGAS) can recognize DNA to trigger activation of stimulator of interferon gene (STING)-mediated innate immunity. STING, first reported by Hiroki Ishikawa and Glen Barber in 2008, is an endoplasmic reticulum adaptor protein critical for innate immune signaling processes (Ishikawa and Barber, 2008). Subsequently, STING was found to mediate IFN-I signaling through IRF3 (Zhong et al., 2008). Then, dimerization of STING was found to be critical for the activation of subsequent downstream signaling (Sun et al., 2009). Finally, it was reported that cGAS is a cytoplasmic DNA sensor that activates STING-mediated antiviral immunity by producing the second messenger cGAMP (Sun et al., 2013; Wu et al., 2013).

As a dangerous stressor, the released DNA is captured by numerous DNA receptors that belong to PRRs in the cell to trigger the innate immune response. For example, interferon-inducible protein 16 (IFI16), as an intracellular DNA sensor and interferon-inducible gene (ISG) induced by IFN-I, mediate STING-dependent antiviral responses (Unterholzner et al., 2010). However, STING can directly interact with IFI16 and facilitate IFI16 degradation *via* the ubiquitin-proteasome pathway by recruiting the E3 ligase triple structural domain protein 21 (TRIM21) (Li et al., 2019), which implies that STING can negatively regulate IFI16 to avoid overactivation of STING. In addition, Sox2 recognizes microbial DNA to activate the complex of kinase TGF-β-activated kinase 1 (TAK1) and its binding partner TAB2, leading to the activation of nuclear transcription factor-κB (NF-κB) and activating protein-1 (AP-1) (Xia et al., 2015). DDX41, a member of the DEXDc family of helicases, also triggers STING-mediated IFN-I production by sensing DNA in the cytoplasm (Zhang et al., 2011). Moreover, TLR9 and absent in melanoma 2 (AIM2) also sense DNA in the cytoplasm to induce innate immune responses (Hornung et al., 2009; West and Shadel, 2017). In addition to the above-mentioned DNA sensors, RNA polymerase III synthesizes 5’-ppp RNA from the poly(dA-dT) template to induce IFN-β through the retinoic acid-induced gene I (RIG-I) pathway (Chiu et al., 2009). However, the DNA sensor cGAS plays a crucial role in the process of the innate immune response. It has been demonstrated that the knockout of cGAS in immune cells fails to produce IFN-I and other inflammatory cytokines in response to DNA virus infection (Li et al., 2013). Thus, activation of the cGAS/STING signaling pathway is critical to counteract the virus compared to other DNA receptor signals. However, viruses have evolved multiple strategies to evade elimination by the cGAS/STING signaling. For example, viruses can suppress innate immunity through direct viral targeting of cGAS and STING (Ma and Damania, 2016). Furthermore, the activation of cGAS and STING is regulated by numerous factors (Wan et al., 2020). Notably, whether the virus suppresses cGAS/STING signaling through related regulatory factors deserves further consideration. Understanding the relationship between the virus and the cGAS/STING signaling is crucial for humans to defeat the virus in the future.

## 2 cGAS/STING signaling

cGAS is a cytoplasmic DNA sensor that synthesizes 2′3′-cyclic-GMP-AMP (cGAMP) in a DNA-dependent manner. Double-stranded DNA (dsDNA) can induce an innate immune response through the cGAS/STING signaling pathway (Sun et al., 2013; Wu et al., 2013). Surprisingly, it has been found that in the resting state, cGAS localizes to the plasma membrane *via* the actions of an N-terminal phosphoinositide-binding domain. In contrast, cGAS translocates to the cytoplasm upon DNA stimulation (Barnett et al., 2019). Moreover, cGAS was also found to localize in the nucleus. However, nucleosomes interact with cGAS to form steric hindrance to inhibit cGAS activation by genomic DNA (Pathare et al., 2020). BAF (barrier-to-autointegration factor 1), a chromatin-binding protein, can compete with cGAS for DNA binding to inhibit the formation of DNA-cGAS complexes (Guey et al., 2020). Cia-cGAS, a circular RNA highly expressed in the nucleus, can bind to the DNA sensor cGAS in the nucleus, blocking its synthetase activity. Mechanistically, cia-cGAS harbors a stronger binding affinity to cGAS than DNA (Xia et al., 2018). These studies suggest that multiple mechanisms exist in cells to avoid potentially damaging autoinflammatory responses caused by cGAS sensing of genomic DNA. Notably, in response to DNA stimulation, cGAS in the nucleus is exported into the cytoplasm by its nuclear export signal to sense DNA (Sun et al., 2021). After cGAS captures DNA, it catalyzes the synthesis of cGAMP from ATP and GTP to activate STING. STING contains a transmembrane domain formed by four transmembrane helices, a cytoplasmic ligand-binding domain (LBD), and a C-terminal tail. In the absence of binding to cGAMP, STING forms butterfly-shaped dimers through the ligand-binding domain. cGAMP binds to the cleft at the center of the butterfly-shaped LBD dimer, which induces a conformational change that promotes the STING oligomerization and translocation of Golgi and perinuclear microsomes (Zhang et al., 2013; Luo et al., 2016; Shang et al., 2019). Subsequently, STING recruits and activates TBK1 through its C-terminal tail containing the conserved PLPLRT/SD motif. Activated TBK1 phosphorylates STING at the PLPLRT/SD motif, promoting further recruitment and activation of TBK1, which subsequently allows STING to recruit IRF-3 and promote IRF3 phosphorylation, ultimately leading to the expression of IFN-I (Zhang et al., 2019; Zhao et al., 2019). IFN-I can further exert antiviral functions by stimulating the production of numerous interferon-stimulated genes (ISGs) products, such as IRF1, IFITM3, and HSPE (Schoggins et al., 2015).

In the process of STING activation, the E3 ubiquitin ligase TRIM32 and TRIM56 interact with STING for K63-linked ubiquitination, promoting STING oligomerization and its recruitment of TBK1 (Tsuchida et al., 2010; Zhang et al., 2012). Notably, IRF8 is essential for DNA but not RNA virus-triggered induction of downstream antiviral genes in monocytes. Upon triggering the DNA sensing pathway, IRF8 interacts with STING after phosphorylation at S151, which promotes oligomerization of STING and its recruitment to IRF3 (Luo et al., 2022). Moreover, a recent study found that a small-molecule agonist, compound 53 (C53), binds to a cryptic pocket of the STING transmembrane domain located between the two subunits of the STING dimer, promoting the oligomerization of STING. Furthermore, the coexistence of cGAMP and C53 in cells more stably activates STING than cGAMP alone (Lu et al., 2022). Thus, C53 analogs are likely to be involved in the process of cellular STING activation, which is worthy of further exploration in the future. In addition, activated STING activates NF-κB signaling through the inhibitor of NF-κB kinase (IKK) complex. Mechanistically, STING activates TRIM32 and TRIM56 to mediate the K63-linked ubiquitination of NF-κB-essential modulator (NEMO), which subsequently activates IKKβ to trigger the phosphorylation of inhibitor of NF-κB (IκBα), thereby activating NF-κB signaling. IKKβ can also activate TBK1, and in turn, TBK1 can also activate IKKβ, which forms a positive feedback loop pathway to strengthen cytokine production during cGAS-STING activation. Notably, activation of NF-κB signaling is essential for the production of IFN-I (Cai et al., 2014; Fang et al., 2017). Recent studies have found that STING is transferred to the Golgi apparatus to bind to sulfated glycosaminoglycans (sGAGs), which promotes STING polymerization and activation of the kinase TBK1. Notably, sGAGs in the Golgi apparatus are necessary and sufficient to drive STING polymerization (Fang et al., 2021). Moreover, palmitoylation at Cys88/91 of STING transported to the Golgi apparatus is required for the IFN-I response (Mukai et al., 2016). Furthermore, upon dsDNA stimulation, the autophagy-related gene 9a (Atg9a), located in the Golgi apparatus, interacts with STING to suppress the assembly of STING and TBK1, interfering with the innate immune response (Saitoh et al., 2009). These results suggest that the activity of STING after Golgi translocation is also regulated by numerous factors.

## 3 Regulation of cGAS/STING signaling

### 3.1 Ubiquitination and SUMOylation of cGAS/STING signaling

Numerous studies have found that the activation of key factors in cGAS/STING signaling is regulated by ubiquitination and SUMOylation. For example, the E3 ubiquitin ligase ring finger protein 5 (RNF5) targets STING for degradation by promoting the ubiquitination of the K48 linkage of STING (Zhong et al., 2009). Of note, RNF26 catalyzes K11-linked polyubiquitination of STING at lysine 150, a residue targeted by RNF5 for K48-linked polyubiquitination (Qin et al., 2014). Thus, RNF26 prevents RNF5 from mediating the k48-linked polyubiquitination and degradation of STING, positively regulating the STING signaling. However, further studies found that RNF26 promotes the autophagy degradation of IRF3, and knockdown of RNF26 can potentiate virus-triggered induction of IFN-I (Qin et al., 2014). These results suggest that RNF26 plays a dual role in the cGAS/STING signaling, and its role may be to limit the excessive activation of IFN-I signaling. Besides, it was found that RNF115 catalyzes the K63-linked ubiquitination of STING, whereas it catalyzes the K48-linked ubiquitination of MAVS. RNF115 knockout mice exhibit hyposensitivity to encephalomyocarditis virus (EMCV) infection and hypersensitivity to herpes simplex virus type 1 (HSV-1) infection (Zhang et al., 2020). Thus, RNF115 positively regulates the cGAS/STING signaling triggered by the DNA virus and negatively regulates the RLR signaling induced by the RNA virus. Furthermore, RNF128 interacts with TBK1 and catalyzes the K63-linked polyubiquitination of TBK1 to promote TBK1-mediated antiviral immunity (Song et al., 2016). However, receptor tyrosine kinase human epidermal growth factor receptor 2 (HER2) strongly interacts with STING and recruits protein kinase B1 (AKT1) to phosphorylate TBK1 at Ser511, a crucial step for disrupting the TBK1-STING interaction and K63-linked ubiquitination of TBK1, attenuating STING signaling (Wu et al., 2019). Finally, activation of cGAS/STING signaling is regulated by numerous other ubiquitin-related enzymes (**Table 1**).

**Table 1:** The regulation of ubiquitination, SUMOylation and expression of key molecules in the cGAS-STING pathway.

Functions	Factors	Target	Proposed Mechanism	Reference
Factors promoting cGAS-STING pathway				
De-SUMOylation	SENP7	cGAS	Cleaving SUMO on the K335/372/382	(Cui et al., 2017)
SUMOylation	TRIM38	cGAS STING	Sumoylating to prevent their polyubiquitination and degradation	(Hu et al., 2016)
E3 ubiquitination				
	RNF26	STING	Catalyzing K11-linked polyubiquitination at K150	(Qin et al., 2014)
	RNF115	STING	Catalyzing the K63-linked polyubiquitination	(Zhang et al., 2020)
	RNF128	TBK1	Catalyzing the K63-linked polyubiquitination	(Song et al., 2016)
	RNF185	cGAS	Catalyzing the K27-linked polyubiquitination	(Wang et al., 2017)
	TRIM41	TBK1 NF-κB BCL10	Catalyzing the K63-linked polyubiquitylation of BCL10 to facilitate the activation of TBK1 and NF-κB	(Yu et al., 2021)
	TRIM32	STING	Catalyzing K63-linked polyubiquitination at K20/150/224/236	(Zhang et al., 2012)
	TRIM56	cGAS	Catalyzing the monoubiquitination at K335	(Seo et al., 2018)
	TRAF6	cGAS	Promoting the activation of cGAS by ubiquitinating	(Chen and Chen, 2019)
	TRIM56	STING	Catalyzing K63-linked polyubiquitination at K150	(Tsuchida et al., 2010)
	AMFR INSIG1	STING	Catalyzing the K27-linked polyubiquitination	(Wang et al., 2014)
	MUL1	STING	Catalyzing the ubiquitination of STING at K224	(Ni et al., 2017)
	Ubc5	IRF3	Catalyzing the K63-linked polyubiquitination	(Zeng et al., 2009)
Deubiquitination				
	IFN-I	cGAS	TRIM14 induced by IFN-I to recruits USP14 to cleave the K48-linked polyubiquitination chain	(Chen et al., 2016)
	EIF3S5	STING	Cleaving the K48-linked polyubiquitination chain	(Luo et al., 2016)
	OTUD5	STING	Cleaving the K48-linked polyubiquitination chain	(Guo et al., 2021)
	USP18	STING	Recruiting USP20 to Remove K48-Linked ubiquitination	(Zhang et al., 2016)
	USP20	STING	Removing K48-linked ubiquitination	(Zhang et al., 2019)
	USP21	STING	Cleaving K27/63-linked polyubiquitin chain	(Chen et al., 2017)
	USP27X	cGAS	Cleaving the K48-linked polyubiquitination chain	(Guo et al., 2019)
	USP29	cGAS	Removing the K48-linked polyubiquitin chains	(Zhang et al., 2020)
	PSMD14	IRF3	Cleaving the K27-linked polyubiquitination at K313 to prevent IRF3 from autophagic degradation	(Wu et al., 2021)
	CYLD	STING	Removing the K48-linked polyubiquitin chains	(Zhang et al., 2018)
Factors inhibiting cGAS-STING pathway				
De-SUMOylation				
	SENP2	cGAS STING	Desumoylating to allow K48-linked ubiquitination	(Hu et al., 2016)
	SENP2	IRF3	Desumoylating to allow K48-linked ubiquitination	(Ran et al., 2011)
E3 ubiquitination				
	RNF178	cGAS	Catalyzing the K63-linked ubiquitination at K411	(Yang et al., 2022)
	RNF90	STING	Catalyzing the K48-linked ubiquitination	(Yang et al., 2020)
	RNF5	STING	Catalyzing the K48-linked ubiquitination at K150	(Zhong et al., 2009)
	TRIM29	STING	Catalyzing the K48-linked ubiquitination	(Xing et al., 2017)
	ASB8	TBK1	Catalyzing the K48-linked ubiquitination	(Guo et al., 2020)
	TRIP	TBK1	Catalyzing the K48-linked ubiquitination	(Zhang et al., 2012)
	SOCS3	TBK1	Catalyzing the K48-linked ubiquitination	(Liu et al., 2015)
	THOC7	TBK1	Catalyzing the K48-linked ubiquitination	(He et al., 2019)
	c-Cbl	IRF3	Catalyzing the K48-linked ubiquitination	(Zhao et al., 2016)
	TRIM26	IRF3	Catalyzing the K48-linked ubiquitination	(Wang et al., 2015)
	RBCK1	IRF3	Catalyzing the K48-linked ubiquitination	(Zhang et al., 2008)
	FoxO1	IRF3	Catalyzing the K48-linked ubiquitination	(Lei et al., 2013)
	TRIM21	IRF3	Catalyzing the K48-linked ubiquitination	(Higgs et al., 2008)
	Pin1	IRF3	Catalyzing the K48-linked ubiquitination	(Saitoh et al., 2006)
	RAUL	IRF3 IRF7	Catalyzing the K48-linked ubiquitination	(Yu and Hayward, 2010)
	TRIM30α	STING	Catalyzing the K48-linked ubiquitination	(Wang et al., 2015)
Deubiquitination				
	USP13	STING	Cleaving the K27-linked polyubiquitination chain	(Sun et al., 2017)
	USP35	STING	Cleaving the K6-, K11-, K27-, K29- or K63-linked polyubiquitination chain	(Zhang et al., 2021)
	USP49	STING	Cleaving the K63-linked polyubiquitination chain	(Ye et al., 2019)
	MYSM1	STING	Cleaving the K63-linked polyubiquitination chain	(Tian et al., 2020)
	OTULIN	NF-κB IFN-I	Suppressing the linear ubiquitination on the immunoproteasome subunit; NF-κB signaling↓ IFN-I signaling↓	(Tao et al., 2021)
	HER2	TBK1	Phosphorylating TBK1 at Ser511 to disrupt the TBK1-STING interaction and K63-linked ubiquitination of TBK1	(Wu et al., 2019)

### 3.2 Other regulation of cGAS/STING signaling

#### 3.2.1 cGAS

In addition to the ubiquitination and sumoylation of cGAS, it has been found that β-arrestin 2 can strengthen the binding of dsDNA to cGAS to enhance cGAMP production by interacting with cGAS, which enhances STING-mediated innate immune responses (Zhang et al., 2020). The binding of DNA to cGAS can robustly induce the phase transition to liquidlike droplets, which function as microreactors in which the enzyme and reactants are highly concentrated to enhance the production of cGAMP. Furthermore, Zn2+ enhances the activity of cGAS by promoting cGAS-DNA phase separation (Du and Chen, 2018). Notably, liquid-liquid separation (LLPS) is a physicochemical process that governs the formation of membraneless condensates, allowing macromolecules, such as proteins and nucleic acids, to condense into dense phases (Peng et al., 2021). The LLPS formed between DNA and cGAS is beneficial to the combination of cGAS and DNA, in which this process is positively regulated by GTPase-activating protein-(SH3 domain)-binding protein 1 (G3BP1). It has been found that G3BP1 engages cGAS in a condensed state, which promotes DNA-induced LLPS and activation of cGAS (Zhao et al., 2021). In addition, recent studies have found that the nuclear-localized heterogeneous nuclear ribonucleoprotein A2B1 (hnRNPA2B1) senses viral DNA and is then translocated into the cytoplasm to activate the TBK1-mediated antiviral signaling. Moreover, hnRNPA2B1 promotes N6-methyladenosine (m6A) modification and nucleocytoplasmic trafficking of cGAS, IFI16, and STING mRNAs, which further potentiate cytoplasmic TBK1 and IRF3 activation mediated by these factors (Wang et al., 2019). Notably, cGAS is also negatively regulated by other DNA sensors. For example, DNA-dependent protein kinase (DNA-PK), a PRR located in the cytoplasm, is a heterotrimeric complex composed of Ku70/Ku80 heterodimers and the catalytic subunit DNA- pkcs, which is important in the innate immune response to intracellular DNA and DNA virus infection. It has been found that human DNA-PK activates the STING-independent DNA sensing pathway (SIDSP) to trigger heat shock protein A8 (HSPA8) and IRF3 phosphorylation, which drives a robust and broad antiviral response. However, this process is not present in mouse cells (Burleigh et al., 2020), which also suggests a large difference between innate immune signaling between species. Notably, DNA-PK negatively regulates cGAS/STING signaling in both humans and mice. It has been found that DNA-PK phosphorylates cGAS and suppresses its enzymatic activity to decrease cGAMP synthesis. Moreover, DNA-PK deficiency in mice potentiates cGAS-mediated antiviral innate immunity and restricts viral replication (Sun et al., 2020). Similarly, the activation of DNA-PK in human cells can also inhibit the activation of cGAS/STING signaling. Poly(ADP-ribose) polymerase 1 (PARP1), a critical nuclear sensor of DNA damage, maintains genomic integrity. Notably, PARP1 is also engaged in the regulation of host innate immunity. More recently, studies have revealed that activated DNA-PK in HeLa cells phosphorylates PARP1 at Thr594, thus triggering the cytoplasmic translocation of PARP1. Then, cytosolic PARP1 directly interacts with and PARylates cGAS at Asp191, which impairs its DNA-binding ability and inhibits antiviral immunity (Wang et al., 2022). The palmitoyltransferase ZDHHC18 catalyzes the palmitoylation of cGAS at C474 to restrict the interaction between cGAS and DNA (Shi et al., 2022). The glutamylation and deglutamylation of cGAS are also intimately related to its activity. Tubulin tyrosine ligase-like 6 (TTLL6)-mediated polyglutamylation of cGAS also impedes its DNA-binding ability, and TTLL4-mediated monoglutamylation of cGAS blocks its synthase activity. Conversely, cytosolic carboxypeptidase CCP6 and CCP5 can remove cGAS polyglutamylation and cGAS monoglutamylation, respectively, restoring cGAS activation (Xia et al., 2016).

Except for the previously mentioned DNA-PK negative regulation of cGAS/STING signaling by targeting cGAS, the DNA sensor AIM2 also curbs cGAS-driven innate immunity. K+ efflux was found to be sufficient to inhibit cGAS-dependent IFN-β responses, but gasdermin D activated by AIM2 inflammasomes restrains cGAS-driven IFN-β production by promoting cellular K+ efflux (Banerjee et al., 2018). These findings imply that activation of other DNA receptors can inhibit cGAS-mediated antiviral immunity, and its significance may lie in avoiding the excessive activation of its innate immune signals. Additionally, miR-23a/b dampens cGAS-mediated innate immunity and autoimmunity by targeting cGAS (Yu et al., 2021). Furthermore, immune-related GTPase M (IRGM) interacts with cGAS and RIG-I to mediate their p62-dependent autophagy degradation. Moreover, it also promotes mitophagy to decrease the accumulation of mitochondrial DAMPs, which restrains interferon signaling (Jena et al., 2020).

#### 3.2.2 STING

Tri-methylation on histone H3 lysine 4 (H3K4me3) strongly correlates with active transcription. However, histone H3K4 lysine demethylases KDM5 suppresses the expression of STING by binding to the promoter of STING and maintaining a low level of H3K4me3, thereby inhibiting cGAS/STING-mediated innate immune response (Wu et al., 2018). cGAMP binds to and activates the adaptor protein STING, leading to IFN-I production and antiviral response. However, the activation of STING is derived not only from the production of intracellular cGAMP but also from the import of extracellular cGAMP. Firstly, cGAMP produced upon HSV-1 infection can be transported to bystander cells *via* the LRRC8A/LRRC8E-containing volume-regulated anion channels (VRACs) to contribute to antiviral IFN response (Zhou et al., 2020). Secondly, the transport of extracellular cGAMP into cells is also dependent on the reduced folate carrier solute carrier family 19 member 1(SLC19A1) (Luteijn et al., 2020). Thirdly, recent studies have found that SLC46A2 is the dominant cGAMP importer in human monocyte-derived macrophages (Cordova et al., 2021). However, the ectonucleotidase ENPP1 can selectively hydrolyze extracellular cGAMP (Li et al., 2021). Therefore, antiviral immune responses can be significantly enhanced by targeting extracellular ENPP1 expression or enhancing the levels of these cGAMP transporters upon viral infection. Surprisingly, recent studies have found that plasmatic membrane STING (pmSTING), an alternatively spliced STING isoform, directly senses extracellular cGAMP to mediate TBK1-mediated antiviral immunity (Li et al., 2022). Furthermore, STING restricts HSV-1 infection independently of IFN signaling (Wu et al., 2020), although the exact mechanism remains unclear. These findings delineate that STING plays a central role in the antiviral immune response. Promoting STING expression and its activation may be the key to defense against viral infection.

The transport of STING from the ER to the Golgi apparatus and perinuclear microsome is critical for its activation of downstream signaling. This process is also positively regulated by many factors. Studies have found that YIPF5, a member of the Yip family (YIPF), recruits STING to coat protein complex II (COPII)-coated vesicles, which facilitates STING trafficking from the ER to the Golgi apparatus (Ran et al., 2019). In addition, the transmembrane emp24 protein transport domain containing 2 (TMED2) interacts with STING to potentiate its activation by reinforcing its dimerization and facilitating its trafficking from the ER to the perinuclear microsome (Sun et al., 2018). Moreover, iRhom2 recruits the translocon-associated protein TRAP-β into the STING complex to facilitate the trafficking of STING from the ER to the perinuclear microsome (Luo et al., 2016). Notably, glutathione peroxidase 4 (GPX4), which maintains redox homeostasis of lipids, is critical for promoting the Golgi translocation of STING and activation of STING signaling. It was found that GPX4 deficiency increases the production of lipid peroxidation, leading to the STING carbonylation at C88 and inhibiting its transport from the ER to the Golgi complex (Jia et al., 2020). Furthermore, transmembrane protein 120A (TMEM120A), a newly discovered antiviral factor, interacts with STING to promote the translocation of STING from the ER to the Golgi apparatus (Li et al., 2022). There are likewise negative regulators in this process. It has been found that mytubin-related protein (MTMR) 3 and MTMR4, members of protein tyrosine phosphatases, disrupt the transport of STING from ER to Golgi apparatus by the production of phosphatidylinositol 3-phosphate (PtdIns3P) (Dewi et al., 2019). In addition, Ca2+ sensor stromal interaction molecule 1 (STIM1) can interact with STING to retain it in the ER membrane (Srikanth et al., 2019). Notably, excessive intracellular accumulation of cGAMP in cells triggers phase separation of ER-associated STING to form the condensate with organized membranous structures, which insulates STING-TBK1 from IRF3, thus preventing overactivation of innate immunity (Yu et al., 2021).

More recently, it was also found that the epidermal growth factor receptor (EGFR) binds STING and mediates its Tyr245 phosphorylation, which is required for IRF3 activation (Wang et al., 2020), suggesting that the phosphorylation of Tyr245 of STING is the key to its activation of downstream antiviral signals. However, many other factors negatively regulate the activity of STING. For example, the protein phosphatase 6 catalytic subunit (PPP6C) negatively regulates the cGAS-STING pathway by removing STING phosphorylation, which reduces IRF3 activation but not NF-κB activation (Ni et al., 2020). Furthermore, PPM1G, a protein phosphatase, can also dephosphorylate STING and MAVS to inhibit STING- and MAVS-mediated IFNβ production (Yu et al., 2020). Current studies have found multiple autophagy-lysosomal degradation pathways of STING, whose significance may be in avoiding excessive activation of STING signaling. For example, the lysosomal membrane protein Niemann-Pick type C1 (NPC1) interacts with STING and recruits it to the lysosome for degradation (Chu et al., 2021). Phosphorylation of S366 of STING favors the activation of downstream signaling. However, cyclic dinucleotides (CDNs) can initially activate the STING function. They subsequently trigger the phosphorylation of STING at S366 to inhibit the activity of IRF3 and subsequent lysosomal degradation of STING, which forms a negative-feedback control of STING activity to avoid the inflammatory disorder (Konno et al., 2013). Thus, phosphorylation of STING S366 is a hallmark of cGAS/STING signaling activation and subsequent degradation. In addition to inducing phosphorylation of STING to activate downstream signaling, TBK1 activated by DNA sensing also phosphorylates the selective autophagy receptor p62, which promotes autophagic degradation of STING and exerts negative control of the cGAS/STING pathway (Prabakaran et al., 2018). Thus, P62 exerts negative control of innate responses to DNA virus infection. These studies suggest that targeting these factors that negatively regulate STING are the potential targets for antipathogen treatment.

#### 3.2.3 TBK1

Activation of TBK1 can further strengthen the antiviral immune response. TBK1 can activate methyltransferase-like 3 (METTL3), the core m6A methyltransferase, *via* two pathways to enhance antiviral signaling. On the one hand, TBK1 phosphorylates METTL3 at serine 67 to facilitate the interaction of METTL3 with the translational complex, which is required for enhancing protein translation, thus strengthening antiviral responses. On the other hand, TBK1 can promote the METTL3-mediated m6A modification to protect IRF3 mRNA from degradation. Furthermore, induction of IFN-I is severely impaired in METTL3-deficient cells (Chen et al., 2022), suggesting that TBK1-mediated METTL3 activation is vital in antiviral immunity. The protein arginine methyl transferase 1 (PRMT1), a type I protein arginine methyltransferase, catalyzes asymmetric methylation of TBK1 to enhance TBK1 oligomerization and phosphorylation, strengthening IFN-I production (Yan et al., 2021). However, the phosphatase Cdc25A interacts with TBK1 and decreases the phosphorylation of TBK1, which reduces the IRF3-mediated generation of IFN-I (Qi et al., 2018). In addition, the neuropeptide hormone kisspeptin secreted by the hypothalamus and pituitary gland binds to G-protein coupled receptor 54 (GPR54) to recruit calcineurin and increase its phosphatase activity, which dephosphorylates TBK1 to restrict antiviral innate immune response (Huang et al., 2018). It has been observed that lncRNA-GM can inhibit the S-glutathionylation of TBK1 to enhance the activity of TBK1 and the downstream production of antiviral mediators (Wang et al., 2020). Furthermore, S-nitrosoglutathione reductase (GSNOR) enhances the TBK1 activity and promotes innate immunity by restricting TBK1 cysteine S-nitrosation at the Cys423 (Liu et al., 2021). Therefore, enhancing the expression of TBK1-related activators and targeting related repressors may be the key to the future treatment of viral infections.

#### 3.2.4 IRF3 and IFN-I

The downstream signaling of the cGAS/STING pathway can, in turn, enhance cGAS/STING signaling and antiviral immune response. For example, the IFN-I can further activate IRF3. Mechanistically, in resting cells, IRF3 is bound to suppressor protein Flightless-1 (Fli-1), which keeps its inactive state. Upon infection, lncRNA-ISIR, an IRF3-binding lncRNA, is induced by IFN-I, which associates with IRF3 to prevent Fli-1 binding with IRF3, thus facilitating IRF3 activation (Xu et al., 2021). Notably, the activation of cGAS can activate IRF3 by non-canonical pathways. It was found that nuclear cGAS also recruits protein arginine methyltransferase 5 (PRMT5) to catalyze the symmetric dimethylation of histone H3 arginine 2 at Ifnb and Ifna4 promoters, which facilitates IRF3 to perform its transcription functions, enhancing the innate antiviral immunity (Cui et al., 2020). Furthermore, CREB-binding protein (CBP) is a histone acetyltransferase, which plays a crucial role in transcriptional regulation. It interacts with IRF3 to enhance cGAS/STING-mediated IFN-β production (Lu et al., 2021). However, IRF3 acetylation can reduce its transcriptional activity. It has been found that lysine acetyltransferase 8 (KAT8) interacts with IRF3 and mediates IRF3 acetylation at K359 to suppress the transcriptional activity of IRF3 (Huai et al., 2019). Moreover, the activated caspase-3 can cleave cGAS, mitochondrial antiviral signaling protein (MAVS), and IRF3 to prevent the production of IFN-I during virus infection-caused apoptosis (Ning et al., 2019).

**Figure 1:**
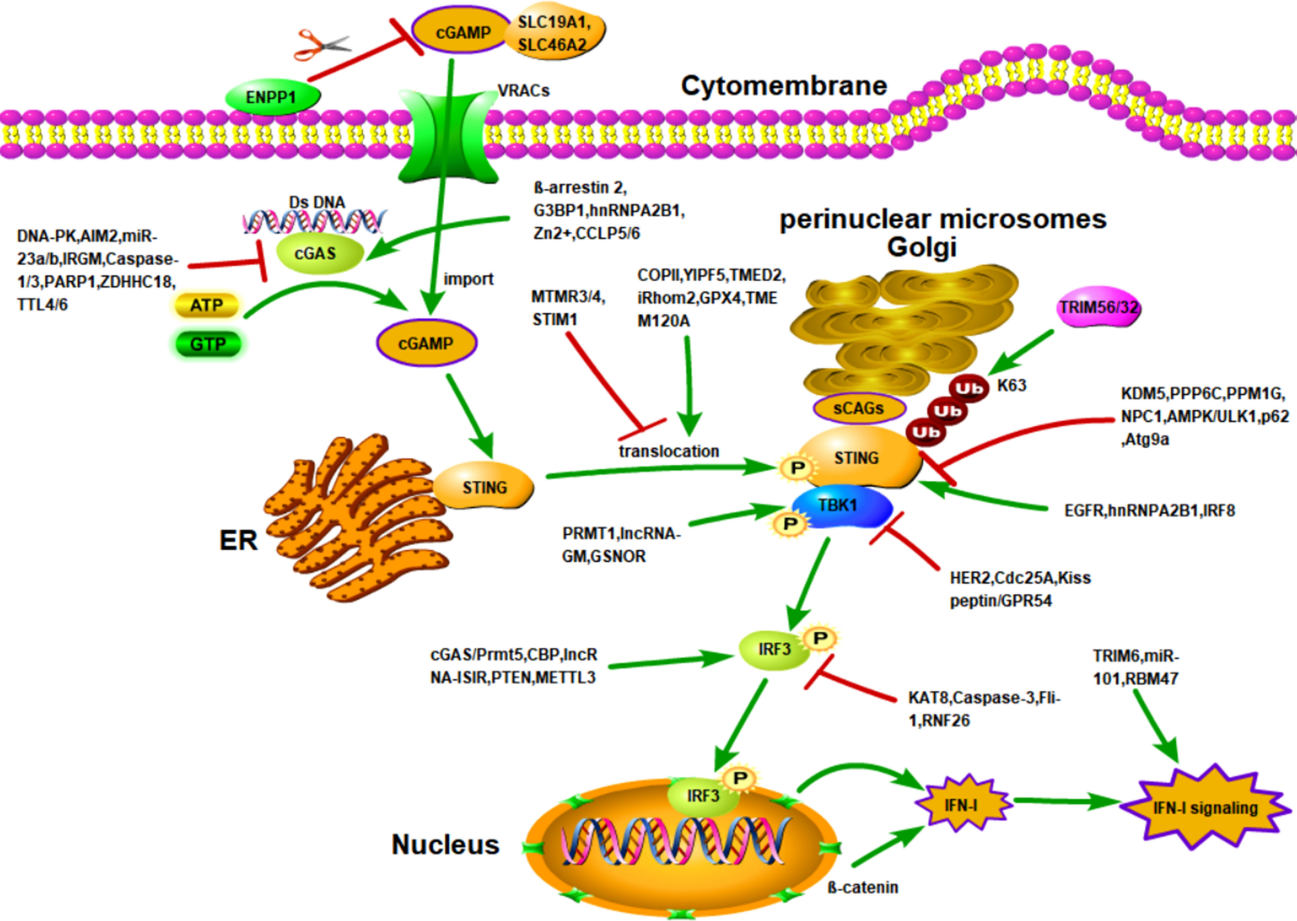
Activation of cGAS/STING signaling and regulation of cGAS/STING signaling by other molecules. Activation of STING is dependent on the presence of intracellular cGAMP. There are two sources of intracellular cGAMP. First, upon binding to DNA, cGAS is activated to catalyze the synthesis of intracellular cGAMP. Second, extracellular cGAMP is transported into the cell through importers. After cGAMP binds to STING, STING is translocated to the Golgi apparatus to bind sCAGs and is linked to the K63-linked ubiquitination by TRIM32 and TIRM56, which induces STING to recruit TBK1 and activate IRF3-induced IFN-I production. The cGAS/STING cascade is controlled by various factors. For example, DNA-PK, AIM2, miR-23a/b, etc. reduce the activity and expression of cGAS through different mechanisms. However, G3BP1, β-arrestin 2, and hnRNPA2B1 enhance the activity and expression of cGAS through different mechanisms. In addition, the entry of extracellular cGAMP into cells is affected by the trafficking of importers SLC19A1 and SLC46A2, as well as by VRACs and ENPP1. Numerous molecules also regulate the translocation of STING. For example, COPII, YIPF5i, Rhom2, etc., enhance the transport of STING from the ER to the Golgi and perinuclear microsomes. However, MTMR3/4 and STIM1 inhibited the translocation of STING. In addition, the activation of STING are also positively and negatively regulated by various factors, such as KDM5, PPP6C, and EGFR.

Numerous factors promote downstream IFN-I signaling (**Figure 1**). Firstly, TRIM6 and the E2 conjugase UbE2K synthesize unanchored K48-linked ubiquitin chains, stimulating IKKϵ for subsequent signal transducer and activator of transcription 1 (STAT1) phosphorylation and IFN-I signaling (Rajsbaum et al., 2014). Meanwhile, VAMP8, a vesicle-associated-membrane protein, is a novel IFN-I signaling regulatory protein whose expression and function depend on the activity of TRIM6 (van Tol et al., 2020). Hence, TRIM6 is critical for the activation of IFN-I signaling. Secondly, miR-101 significantly strengthens IFN-I production and IFN-I-mediated antiviral innate defense to suppress the replication of feline herpesvirus 1 (FHV-1) by targeting the suppressor of cytokine signaling factor 5 (SOCS5), a negative regulator of the JAK-STAT pathway (Zhang et al., 2020). Thirdly, β-catenin facilitates activation of the cGAS/STING signaling pathway by translocating to the nucleus and then up-regulating the IFN-β promoter activity. However, HSV-1 US3 promotes the hyperphosphorylation of β-catenin at Thr556 to block its nuclear translocation, which subverts innate antiviral immunity (You et al., 2020). Finally, the RNA binding protein RBM47, an ISG, can be up-regulated by IFN-I stimulation. It has been found that RBM47 binds to the 3’UTR of IFNAR1 mRNA to increase mRNA stability, which retards the degradation of IFNAR1 and potentiates innate immunity (Wang et al., 2021).

## 4 The hijacking of cGAS/STING signaling by viruses

Based on the above results, the cGAS-STING signal transduction is very complicated, in which the process is based on various factors. However, the virus has also evolved numerous strategies to suppress the cGAS/STING signaling (**Table 2**). cGAS can induce the activation of downstream signals by recognizing DNA. Therefore, DNA virus infection will cause its DNA fragments to be recognized by cGAS, which promotes activation of the cGAS/STING signaling pathway. Unsurprisingly, DNA viruses have also evolved various mechanisms to counteract the host cGAS/STING pathway. However, surprisingly, RNA virus infection can also trigger cGAS-mediated innate immunity. For example, dengue virus (DENV) and west nile virus (WNV) also initiate cGAS-mediated innate immunity in mitochondrial DNA (mtDNA)-dependent manner (Schoggins et al., 2014; Aguirre et al., 2017). Recent studies have also found that mitochondrial stress caused by measles virus (MeV) infection induces increased cytosolic mtDNA into the cytosol, where mtDNA is captured by cGAS and causes consequent priming of the DNA sensing pathway (Sato et al., 2021). Moreover, activation of cGAS relies on its recognition of cytoplasmic chromatin DNA shuttled from the nucleus as a result of cell-to-cell fusion upon SARS-CoV-2 infection (Zhou et al., 2021). Thus, the activation of cGAS triggered by RNA viruses not only depends on the release of mtDNA but also on chromosomal DNA. Similarly, RNA viruses also utilize multiple strategies to escape cGAS/STING signaling. For example, although the influenza A virus M2 protein triggers mtDNA translocation into the cytosol and mediates the antiviral immune response, the nonstructural protein 1 (NS1) of influenza A virus associates with mtDNA to evade STING-dependent antiviral immunity (Moriyama et al., 2019).

**Table 2:** Hijacking of cGAS/STING signaling by viruses.

Virus Family	Virus	Viral Protein	Target	Proposed Mechanism	Reference
Herpesviridae					
	HSV-1	UL37	cGAS	Deamidation of cGAS	(Zhang et al., 2018)
	HSV-1	?	cGAS	Degradation of β-arrestin 2 to inhibit the activation of cGAS	(Zhang et al., 2020)
	HSV-1	?	DNA-PK	PARylating cGAS to inhibit its DNA-binding ability	(Wang et al., 2022)
	HSV-1	UL41	cGAS	Degradation of cGAS	(Su and Zheng, 2017)
	γ-HSV/α-HSV	ORF52/VP22	cGAS	Restriction of cGAS-DNA phase separation	(Xu et al., 2021)
	HSV-1	?	TRIM32 TRIM41	Inhibiting the activation of STING and TBK1	(Yu et al., 2021)
	HSV-1	?	MYSM1	MYSM1 interacts with STING and cleaves STING K63-linked ubiquitination	(Tian et al., 2020)
	HSV-1	?	TBK1	Decreasing lncRNA-GM to increase S-glutathionylation of TBK1	(Wang et al., 2020)
	HSV-1	?	TBK1	Promoting TBK1 s-nitrosation	(Liu et al., 2021)
	HSV-1	VP24	TBK1; IRF3	Abrogating the interaction between TBK1 and IRF3	(Zhang et al., 2016)
	HSV-1	US3	IRF3	Hyperphosphorylation of IRF3 at Ser175 to prevent IRF3 activation	(Wang et al., 2013)
	HSV-1	US3	IFN-β	Interacting with β-catenin; IFN-β↓	(You et al., 2020)
	HSV-1	VP1-2	STING	Cleaving the K63-linked polyubiquitination chain of STING	(Bodda et al., 2020)
	HSV-1	VP16	NF-κB; IRF3 and CBP	Inhibiting NF-κB activation; Blocking IRF3 to recruit its coactivator CBP	(Xing et al., 2013)
	HSV-1	UL24	NF-κB	Inhibit the activation of NF-κB; IFN-β↓	(Xu et al., 2017)
	HSV-1	UL36	NF-κB	Deubiquitinate IκBα to restrict NF-κB activation; IFN-β↓	(Ye et al., 2017)
	HSV-1	UL46	STING; TBK1	STING protein↓; IFN-β and ISG56 mRNA↓	(Deschamps and Kalamvoki, 2017)
	HSV-2	ICP27	IRF3	Inhibiting phosphorylation and nuclear translocation of IRF3	(Guan et al., 2019)
	HSV-2	US1	IRF3	Interacting with DNA binding domain of IRF-3	(Zhang et al., 2015)
	HSV-2	ICP22	IFN-I Signaling	Degradation of STAT1, STAT2, IRF9 and ISGF3	(Zhang et al., 2020)
	MDV	RLORF4	p65 and p50	Interfering with their nuclear translocation	(Liu et al., 2019)
	MDV	VP23	TBK1	Inhibiting the interaction between IRF7 and TBK1;Suppressing IRF7 activation	(Gao et al., 2019)
	KSHV	ORF33	STING PPM1G	Reducing STING phosphorylation	(Yu et al., 2020)
	KSHV	ORF48	PPP6C	Reducing STING phosphorylation	(Ni et al., 2020)
	KSHV	ORF50	USP7	Degradation of IRF3 and IRF7	(Yu and Hayward, 2010)
	KSHV	ORF52	cGAS	Disrupting cGAS binding to DNA	(Wu et al., 2015)
	KSHV	LANA	cGAS	Interacting with cGAS to inhibit the phosphorylation of TBK1 and IRF3	(Zhang et al., 2016)
	KSHV	LANA	NF-κB	Inhibiting the NF-κB activation	(Mariggio et al., 2017)
	KSHV	vIRF1	STING	Reducing STING phosphorylation; Inhibiting STING binding to TBK1	(Ma et al., 2015)
	HCMV	pUL83	cGAS	Inhibiting enzymatic activity of cGAS	(Biolatti et al., 2018)
	HCMV	UL31	cGAS	Inhibiting DNA binding to cGAS	(Huang et al., 2018)
	HCMV	UL42	cGAS/STING	Inhibiting DNA binding to cGAS and translocation of MITA	(Fu et al., 2019)
	HCMV	UL82	STING	Interacting with STING to inhibit the translocation of STING	(Fu et al., 2017)
	HCMV	UL94	STING	Disrupting the dimerization and translocation of STING	(Zou et al., 2020)
	EBV	?	TRIM29	Degradation of STING	(Xing et al., 2017)
	EBV	Zta	TBK1 and IRF3	Inhibiting TBK1 and IRF3 activation	(Wong et al., 2017)
	PRV	UL13	IRF3	Phosphorylating IRF3 to attenuate the binding of IRF3 to the IFN-β promoter	(Bo et al., 2020)
	PRV	gE	IRF3 CBP	IFN-β↓; CBP protein degradation	(Lu et al., 2021)
Coronaviridae					
	HCoV-NL63	PLP2-TM	STING	Interacting with STING and disrupts STING dimers; Suppressing K63-linked polyubiquitination of STING	(Sun et al., 2012) (Clementz et al., 2010)
	MERS-CoV	M protein	TBK1	Suppressing TBK1-dependent phosphorylation and activation of IRF3	(Lui et al., 2016)
	MERS-CoV	ORF4b	TBK1	Interacting with TBK1; IFN-β↓	(Yang et al., 2015)
	SARS-CoV	PLpro-TM	STING	Interacting with STING; Inhibiting K63-linked polyubiquitination of STING	(Chen et al., 2014)
	SARS-CoV-2	M protein	TBK1	Interacting with TBK1 to degrade TBK1	(Sui et al., 2021)
	SARS-CoV-2	ORF9b	STING; TBK1	Impeding the phosphorylation and nuclear translocation of IRF3	(Han et al., 2021)
	SARS-CoV-2	ORF3a; 3CL	STING	Impeding the nuclear translocation of IRF3; Inhibiting K63-linked ubiquitination of STING	(Rui et al., 2021)
	SARS-CoV-2	nsp12	IRF3	Preventing the nuclear translocation of IRF3	(Wang et al., 2021)
	SARS-CoV-2	NSP5	IRF3	Preventing the nuclear translocation of IRF3	(Fung et al., 2021)
	PEDV	PLP2	STING	Inhibiting K63-linked polyubiquitination of STING	(Xing et al., 2013)
	PEDV	N protein	TBK1	Inhibiting the interaction between TBK1 and IRF3	(Ding et al., 2014)
	PEDV	nsp1	CBP	CBP protein degradation	(Zhang et al., 2016)
	PEDV	nsp15	TBK1 IRF3	Degrading the RNA levels of TBK1 and IRF3	(Wu et al., 2020)
	PEDV	PLP1	?	Interacting with PCBP2; IFN-β↓	(Zhang et al., 2020)
Poxviridae					
	VACV	?	caspase-3	Activated caspase-3 to cleave cGAS and IRF3	(Ning et al., 2019)
	CPXV/ECTV	?	STING	Inhibiting STING dimerization and phosphorylation	(Georgana et al., 2018)
	VACV	?	TBK1	Decreasing lncRNA-GM to increase S-glutathionylation of TBK1	(Wang et al., 2020)
	VACV	F17	mTOR	mTOR Dysregulation; Suppressing ISG responses.	(Meade et al., 2019)
Papillomaviridae					
	HPV	E7	STING	Interacting with STING	(Lau et al., 2015)
	HPV	E7	IFI16 TRIM21	Degradation of IFI16	(Song et al., 2020)
Adenoviridae					
	Adenovirus	E1A	STING	Interacting with STING	(Lau et al., 2015)
	Adenovirus	E1A	IFN-I Signaling	SuppressingSTAT1 activation	(Look et al., 1998)
Hepadnaviridae					
	HBV	Polymerase	STING	Inhibiting K63-linked ubiquitination of STING	(Liu et al., 2014)
	HBV	?	IRF3	IRF3↓IFN-β↓	(Shravanthi et al., 2015)
	HBV	?	PTEN	Inhibiting IRF-3 nuclear translocation	(Kim et al., 2021)
Orthomyxoviridae					
	Influenza A virus	NS1	mtDNA	Inhibition of mtDNA release and binding to cGAS	(Moriyama et al., 2019)
Flaviviridae					
	DENV	NS2B	cGAS	Degradation of cGAS	(Aguirre et al., 2017)
	DTMUV	NS2A	STING; TBK1	Impairing STING-STING binding; Reducing TBK1 phosphorylation	(Zhang et al., 2020)
	DTMUV	NS2B3	STING	Cleaving STING	(Wu et al., 2019)
	HCV	NS4B	STING	Blocking interaction between STING and STING	(Nitta et al., 2013)
	HCV	NS4B	STING	Suppressing STING Accumulation	(Yi et al., 2016)
	ZIKV	NS1	cGAS	Inhibiting the proteasomal degradation of caspase-1 to cleave cGAS	(Zheng et al., 2018)
	ZIKV	NS2B3	STING	Cleaving STING	(Ding et al., 2018)
Paramyxoviridae					
	SeV	?	caspase-3	Activated caspase-3 to cleave cGAS and IRF3	(Ning et al., 2019)
	SeV	?	TBK1	Decreasing lncRNA-GM to increase S-glutathionylation of TBK1	(Wang et al., 2020)
	SeV	?	ASB8	Promoting the interaction of ASB8 with TBK1 to degrade TBK1	(Guo et al., 2020)
	SeV	?	TBK1	Promoting TBK1 s-nitrosation	(Liu et al., 2021)
	SeV	?	IRF3	Degradation of IRF3	(Wang et al., 2015)
	SeV	?	PSMD14	Degradation of IRF3	(Wu et al., 2021)
	SeV	?	RBCK1	Increased RBCK1 to degrade IRF3	(Zhang et al., 2008)
	NiV	matrix protein	IFN-I signaling	Interacting with TRIM6 to suppress TRIM6-mediated IFN-I signaling	(Bharaj et al., 2016)
Picornaviridae					
	FMDV	Lpro	TBK1	Cleaving TBK1	(Visser et al., 2020)
	FMDV	Lpro	IRF3/7	Decreasing the protein level of IFR3/7	(Wang et al., 2010)
	FMDV	Lpro	IFN-I; ISG	Decreasing the protein level of IFN-I and ISG	(Medina et al., 2020)
	mengovirus	L proteins	IFN-I	Inhibiting IFN-I gene transcription	(Hato et al., 2010)
	FMDV	Lpro	p65	Degradation of p65	(De Los Santos et al., 2007)
Retroviridae					
	HIV-1	capsid	cGAS	Disrupting cGAS binding to viral DNA	(Cingoz and Goff, 2019)
	HIV-2/SIV	Vpx	STING	Inhibiting STING activation; Interfering with the nuclear translocation of NF-κB	(Su et al., 2019)
	HTLV-1	Tax	STING	Inhibiting K63-linked ubiquitination of STING and interactions between STING and TBK1	(Wang et al., 2017)
	HTLV-1	Tax	TBK1	Inhibiting IRF3 phosphorylation	(Yuen et al., 2016)
	SIV	?	STING	Including expression of NLRX1	(Barouch et al., 2016)
Rhabdoviridae					
	VSV	?	β-arrestin 2	Degradation of β-arrestin 2 to inhibit the activation of cGAS	(Zhang et al., 2020)
	VSV	?	TRIM32 TRIM41	Inhibiting the activation of STING and TBK1	(Yu et al., 2021)
	VSV	?	Kisspeptin/GPR54 signaling	Dephosphorylating TBK1	(Huang et al., 2018)
	VSV	?	lncRNA-GM	Decreasing lncRNA-GM to increase S-glutathionylation of TBK1	(Wang et al., 2020)
	VSV	?	TRIM26	Degradation of IRF3	(Wang et al., 2015)
	VSV	?	caspase-3	Activated caspase-3 to cleave IRF3	(Ning et al., 2019)
Phenuiviridae					
	HRTV	NSs	TBK1	Blocking the interaction between TBK1 and IRF3	(Ning et al., 2017)
	HRTV	NSs and others	IFN-I signaling	Dampening both STAT2 and STAT1 activities	(Feng et al., 2019)
Peribunyaviridae					
	GTV	NSs	TBK1, STAT2	Sequesters TBK1and STAT2 into the viral IBs and FSs.	(Min et al., 2020)
Asfarviridae					
	ASFV	?	STING	Inhibiting the activation of STING; IFN-β↓	(García-Belmonte et al., 2019)
	ASFV	MGF360-11L	TBK1 IRF3	Inhibiting the phosphorylation of TBK1 and IRF3	(Yang et al., 2022)
Parvoviridae					
	FPV	NS2	TBK1	Disrupting the Interaction between STING and TBK1	(Kang et al., 2017)
	HBoV	NP1	IRF-3	Interacting with DNA binding domain of IRF-3	(Zhang et al., 2012)
Arteriviridae	PRRSV/LDV/SHFV	nsp1	CBP	CBP protein degradation	(Han and Yoo, 2014)
Circoviridae	PCV2	Cap and its binding protein gC1qR	cGAS	Catalyzing the K48-linked ubiquitination	(Wang et al., 2021)

### 4.1 DNA viruses

#### 4.1.1 Herpesviridae

Since cGAS activates the STING-dependent antiviral immunity by capturing DNA, various DNA viruses trigger cGAS/STING signaling, including herpes virus, vaccinia virus, and adenovirus (Lam et al., 2014; Dai et al., 2014; Wu et al., 2015). Notably, Herpesviridae family viruses can directly inhibit the activation of cGAS/STING signaling through their tegument proteins (**Table 2**). For example, the HSV-1 UL37 tegument protein deamidates cGAS, which decreases the ability of cGAS to catalyze cGAMP synthesis, contributing to virus replication (Zhang et al., 2018). It was recently found that the LLPS formed by cGAS-DNA facilitates the interaction between cGAS and STING and activation of innate immune signaling. However, γ-herpesvirus ORF52 and α-herpesvirus VP22 disrupt the LLPS to alter the innate immune response in favor of viral replication (Xu et al., 2021). Similarly, human cytomegalovirus (HCMV) protein UL31 also directly interacts with cGAS to disassociate DNA from cGAS, suppressing the cGAS enzymatic functions and cGAMP production (Huang et al., 2018). In addition, HSV-1 tegument protein UL41 abrogates the cGAS/STING-mediated DNA sensing pathway by selectively degrading cGAS mRNA to downregulate the expression of cGAS through its RNase activity (Su and Zheng, 2017). Furthermore, the HCMV tegument protein pp65 (pUL83) selectively binds to cGAS and inhibits its enzymatic activity (Biolatti et al., 2018). It has been mentioned above that the functional activity of cGAS is regulated by numerous factors. Therefore, viruses likely affect the function of cGAS through these pathways. Indeed, HSV-1 infection induces host cell DNA damage that promotes DNA-PK-dependent inhibition of cGAS activity (Wang et al., 2022). However, whether Herpesviridae inhibits cGAS activity through other related regulatory factors remains unclear.

Of note, many studies have proved that viruses of the herpesvirus family can inhibit the activation of STING through the above approach. Firstly, the expression of MPN domains 1 (MYSM1) is induced upon DNA virus infection. MYSM1 subsequently interacts with STING and cleaves STING K63-linked ubiquitination to suppress cGAS-STING signaling (Tian et al., 2020). Secondly, Kaposi’s sarcoma-associated herpesvirus (KSHV) ORF48 interacts with PPP6C to inhibit STING-dependent IFN-I production by removing the phosphorylation of STING (Ni et al., 2020). In addition, KSHV ORF33 interacts with STING/MAVS and enhances the recruitment of PPM1G to dephosphorylate STING/MAVS, restricting innate immune signaling (Yu et al., 2020). Furthermore, Epstein-Barr virus (EBV), a γ herpes virus, induces TRIM29 to mediate K48-linked ubiquitination of STING for its rapid degradation (Xing et al., 2017). Finally, HSV-1 reduces the expression of TRIM32 and TRIM41 (Yu et al., 2021), suggesting that HSV-1 can inhibit the activation of STING and TBK1 and negatively regulate innate immunity.

Viruses of the herpesvirus family can suppress the activation of TBK1 and IRF3 through the above approach. For example, HSV-1 infection promotes TBK1 S-nitrosation to suppress the innate immune response by inducing reactive nitrogen species (RNS) (Liu et al., 2021), which means that the virus directly utilizes the S-nitrosation of TBK1 to inactivate TBK1. Given that GSNOR inhibits s-nitrosation of TBK1 to enhance innate immunity, it is still unclear whether viral infections can hijack GSNOR to antagonize antiviral immunity. Moreover, pseudorabies virus (PRV) glycoprotein E (gE) degrades CBP to dampen cGAS/STING-mediated IFN-β production (Lu et al., 2021). In addition, HSV-1 VP16 and porcine reproductive and respiratory syndrome virus (PRRSV) nonstructural protein 1 α (nsp1α) can inhibit IFN-I production by interfering with the recruitment of CBP to IRF3 and degrading CBP, respectively (Xing et al., 2013; Han and Yoo, 2014). Thus, CBP may be a common target of herpes viruses, and how to prevent the activity of CBP from being suppressed by the virus may be an essential strategy to fight against herpes virus infections.

Notably, KSHV ORF50 mediates RAUL deubiquitination *via* USP7, thus decreasing the expression of IRF3 and IRF7 (Yu and Hayward, 2010). In addition, the herpes virus can directly inhibit the activation of TBK1 and IRF3 to suppress innate antiviral immunity. For example, EBV lytic infection suppresses IFN-β production. Mechanistically, EBV lytic transactivator Zta decreases the phosphorylation of TBK1 and IRF3 to block the nuclear translocation of IRF3 (Wong et al., 2017). Notably, IRF7 also plays a vital role in the innate immunity mediated by cGAS/STING. However, it has been observed that Marek’s disease virus (MDV) VP23 interacts with IRF7 to block its binding to TBK1, which inhibits phosphorylation and nuclear translocation of IRF7 and cGAS/STING-induced IFN-β production (Gao et al., 2019). Importantly, activation of NF-κB signaling is likewise essential for IFN-I production. However, MDV RLORF4 interacts with NF-κB subunits p65 and p50 to inhibit cGAS-STING-mediated NF-κB activation, thereby reducing IFN-β production (Liu et al., 2019).

In addition, HSV-1 protein ICP0, an E3 ubiquitin ligase, blocks the innate immune response induced by DNA-PK (Burleigh et al., 2020). However, the viral protein ICP0 does not appear to suppress innate immunity. It was found that STING is not degraded in wild-type HSV-1-infected cancer-derived HEp-2 or HeLa cells, but it is degraded in cells infected with ICP0 deletion mutant of HSV-1 (Kalamvoki and Roizman, 2014). However, in human embryonic lung cells, STING is not degraded after infection with either wild-type or ICP0 mutant viruses (Kalamvoki and Roizman, 2014). Hence, whether HSV-1 causes the degradation of STING may depend on cell type and context. Furthermore, HSV-1 infection results in a marked decrease in IFI16 protein levels in normal human foreskin fibroblasts (HFFs), normal oral keratinocytes (NOKs), and HeLa cells, but not in U2OS cells (Orzalli et al., 2016), suggesting that whether HSV-1 causes the degradation of IFI16 is also intimately related to cell type.

#### 4.1.2 Hepadnaviridae

Hepatitis B virus (HBV) infection significantly reduces host cell IRF3 expression (Shravanthi et al., 2015). Moreover, HBV polymerase interacts with STING and disrupts the k63-linked ubiquitination of STING through its reverse transcriptase (RT) domains, suppressing IRF3 activation and IFN-β induction (Liu et al., 2014). Furthermore, as a tumor suppressor, phosphatase and tensin homolog (PTEN) is also involved in regulating innate immunity. It dephosphorylates IRF-3 at the Ser97 site to strengthen IRF-3 nuclear translocation, which enhances the IFN signaling pathway. However, HBV significantly increases m6A modification of PTEN mRNA, which contributes to its instability with a corresponding decrease in PTEN protein levels, thus blocking the IRF3 nuclear import and IFN signaling pathway (Kim et al., 2021).

#### 4.1.3 Papillomaviridae and adenoviridae

Both human papillomavirus (HPV) and adenovirus can directly target STING to suppress activation of the cGAS/STING pathway. It has been demonstrated that HPV E7 and adenovirus E1A antagonize the cGAS-STING DNA-sensing pathway by interacting with STING. Further, the LXCXE motif of E7 and E1A is crucial for the blockade of the cGAS/STING signaling (Lau et al., 2015), which suggests that targeting the LXCXE motif is one of the strategies against HPV and adenovirus. Moreover, HPV E7 decreases the IFI16 expression by recruiting TRIM21, evading immune surveillance (Song et al., 2020). Furthermore, deletion of HPV E7 restores the cGAS-STING response (Bortnik et al., 2021). Thus, targeting the HPV viral E7 protein is among the critical approaches to treating HPV infection. Notably, adenovirus E1A can directly engage with STAT1 to block STAT1-dependent gene activation (Look et al., 1998). Therefore, adenovirus E1A can inhibit not only cGAS/STING signaling but also IFN-I-mediated antiviral signaling to escape innate immunity. In addition, human adenovirus E1A blocks the antiviral signaling pathway triggered by DNA-PK (Burleigh et al., 2020). However, one study found that knockdown of either cGAS or STING in Hela cells did not affect adenoviral DNA replication (Lam and Falck-Pedersen, 2014), suggesting that adenovirus replication may be independent of activating cGAS/STING signaling, and the mechanism remains unclear.

#### 4.1.4 Poxviridae

The activated caspase-3 cleaves cGAS and IRF3, a process used by vaccinia virus (VACV) to suppress innate immunity (Ning et al., 2019). Notably, DNA-PK also binds to DNA to induce innate immunity independent of STING. However, VACV C16 directly binds to the Ku heterodimer to block DNA-PK binding to DNA, hampering DNA-PK-dependent DNA sensing (Peters et al., 2013). Moreover, VACV C4 has also been shown to antagonize DNA-PK by binding to Ku and blocking the binding of Ku to DNA, which induces the diminished production of cytokines and chemokines (Scutts et al., 2018). In addition, VACV F17 suppresses ISG responses by dysregulating the host kinase mammalian target of rapamycin (mTOR) (Meade et al., 2019), suggesting that in addition to NF-κB signaling, mTOR signaling is also critical in innate immune responses. These results also show that VACV can suppress innate immunity in various ways. Blocking these pathways is also an essential strategy in treating VACV infection. Furthermore, cowpox virus (CPXV) and ectromelia virus (ECTV) infection prevent STING phosphorylation and dimerization (Georgana et al., 2018), but the specific mechanism remains unclear.

### 4.2 RNA viruses

#### 4.2.1 Flaviviridae

Many viruses in the Flaviviridae family directly target critical factors in the cGAS/STING pathway to suppress innate immunity (**Table 2**). Firstly, the DENV NS2B protein targets cGAS for degradation to avoid the detection of mtDNA upon infection (Aguirre et al., 2017). Secondly, Zika virus (ZIKV) infection triggers NLRP3 inflammasome activation, in which the process is further enhanced by ZIKV NS1 to facilitate its replication. Further, NS1 recruits the deubiquitinase USP8 to cleave K11-linked poly-ubiquitin chains from caspase-1, preventing the proteasomal degradation of caspase-1. Thus, the caspase-1 stabilized by NS1 strengthens the cleavage of cGAS, which attenuates cGAS-mediated IFN-I production and facilitates the immune escape of ZIKV (Zheng et al., 2018). Based on the above results, these suggest that NLRP3 inflammasome activation suppresses cGAS-mediated type I IFN signaling by stabilizing caspase-1, but this process is utilized by ZIKV NS1.

#### 4.2.2 Coronaviridae

SARS-CoV-2 ORF9b interacts with STING and TBK1 to impede the IRF3 phosphorylation and nuclear translocation, thus negatively regulating antiviral immunity and facilitating viral replication (Han et al., 2021). Moreover, both ORF3a and 3CL of SARS-CoV-2 can inhibit the activation of STING signaling. Mechanistically, ORF3a uniquely binds STING, blocking the nuclear accumulation of p65 and inhibiting the NF-κB signaling pathway. SARS-CoV-2 3CL disrupts k63-linked ubiquitination of STING and blocks the STING-dependent downstream signaling (Rui et al., 2021). Human coronavirus (HCoV) NL63 (the membrane-anchored PLP domain, PLP2-TM) has been shown to interact with STING to inhibit STING dimer formation, which is not dependent on PLP2-TM’s deubiquitinase (DUB) activity (Sun et al., 2012). Moreover, PLP2-TM attenuates the K63-linked ubiquitination of STING, which is not dependent on PLP2-TM’s DUB activity (Clementz et al., 2010), suggesting that the interaction of PLP2-TM with STING may be the key to decreasing the K63-linked ubiquitination of STING. However, porcine epidemic diarrhea virus (PEDV) papain-like protease 2 (PLP2) has been reported to repress the K63-linked ubiquitination of STING through its DUB activity (Xing et al., 2013). Therefore, the differences between human and porcine viruses deserve further clarification. PEDV PLP1 interacts with host cell poly(C)-binding protein 2 (PCBP2) to decrease the production of IFN-I and promote the replication of PEDV (Zhang et al., 2020). However, whether this process is associated with inhibition of cGAS/STING signaling remains elusive. In addition, the SARS-CoV membrane-anchored PLpro domain (PLpro-TM) has been reported to inhibit the K63-linked ubiquitination of STING and STING-mediated activation of downstream signaling by associating with STING (Chen et al., 2014). These findings indicate that the papain-like protease domain may be the key to the suppression of innate immune responses by the Coronaviridae family. Notably, it appears that both the viral nucleocapsid (N) and membrane (M) proteins of the Coronaviridae target TBK1. PEDV-encoded nucleocapsid (N) protein has also been shown to interact with TBK1 and sequester the association between TBK1 and IRF3 (Ding et al., 2014). SARS-CoV-2 M protein interacts with TBK1 and induces TBK1 degradation *via* K48-linked ubiquitination (Sui et al., 2021). Furthermore, the M proteins of Middle East respiratory syndrome coronavirus (MERS-CoV) and SARS-CoV have three highly similar conserved N-terminal transmembrane domains and a C-terminal region. Further studies found that the N-terminal transmembrane domain of the MERS-CoV M protein is sufficient to inhibit IFN-I expression, but not the C-terminal domain (Lui et al., 2016), suggesting that the N-terminal transmembrane domain of M protein may primarily involve in the interaction with TBK1 to induce its degradation process. Notably, although IFN-I limits viral reproduction in the early stage of viral infection, a sustained increase in the levels of IFN-I in the late stage of infection is associated with aberrant inflammation and poor clinical outcome. It has been demonstrated that SARS-CoV-2 infection activates cGAS/STING signaling to promote the IFN-I production in endothelial cells through mtDNA release. Inhibition of STING attenuates severe SARS-CoV-2-induced lung inflammation and improves disease outcomes (Domizio et al., 2022). Thus, the activation of cGAS/STING signaling plays a more detrimental role in the severe inflammatory response at the late stage of a viral infection than the beneficial effect of limiting viral replication.

#### 4.2.3 Picornaviridae

The lead protease (Lpro) of foot-and-mouth disease virus (FMDV) can interfere with cGAS/STING signaling through various strategies. Firstly, the Lpro of FMDV cleaves TBK1 to decrease the IFN-I production (Visser et al., 2020). Secondly, the FMDV Lpro also directly reduces the protein expression of IRF-3/7 (Wang et al., 2010). Thirdly, the Lpro of FMDV translocates to the nucleus to stimulate the degradation of nuclear p65, antagonizing the innate immune and inflammatory responses (De Los Santos et al., 2007). Therefore, targeting the Lpro of FMDV may be one of the strategies for treating FMDV infection. In addition, the L protein of mengovirus, a strain of encephalomyocarditis virus, suppresses the antiviral response by inhibiting the transcription of the IFN-I gene (Hato et al., 2010).

#### 4.2.4 Rhabdoviridae

It has been demonstrated that vesicular stomatitis virus (VSV) utilizes a variety of cGAS/STING signal-related regulatory factors to suppress cGAS/STING signaling. First, VSV negatively regulates cGAS-mediated innate immunity by significantly reducing the expression of E3 ubiquitin ligases TRIM32 and TRIM41 (Yu et al., 2021). Secondly, the increased kisspeptin secreted by the hypothalamus and pituitary gland during VSV infection restricts the innate immune response by GPR54/calcineurin axis (Huang et al., 2018). Moreover, VSV infection promotes the degradation of β-arrestin 2 to facilitate immune evasion (Zhang et al., 2020). Furthermore, VSV infection also down-regulates the expression of lncRNA-GM to facilitate VSV escape by disrupting TBK1 activity (Wang et al., 2020). Finally, VSV infection promotes the nuclear translocation of TRIM26 to degrade IRF3 (Wang et al., 2015). It is worth noting that the viral protein on the surface of VSV has not been found to inhibit the cGAS/STING signaling directly, and further exploration is necessary.

#### 4.2.5 Retroviridae

Sensing of HIV and other retroviruses relies on viral reverse transcription and the cellular DNA sensor GAS (Gao et al., 2013). However, studies have shown that THP-1 cells do not lead to significant innate immune induction to HIV-1 infection (Cingoz and Goff, 2019). Mechanistically, the HIV-1 capsid protects viral DNA from the cGAS DNA sensing (Sumner et al., 2020). In addition, NLRX1 interacts with STING to prevent STING-TBK1 interaction and suppress innate immunity (Guo et al., 2016). This function seems to be fully exploited by HIV-1 and simian immunodeficiency virus (SIV) (Guo et al., 2016; Barouch et al., 2016). HIV-2/SIV Vpx can also target STING to suppress cGAS-STING-mediated NF-kB signaling, impeding the induction of innate immune genes (Su et al., 2019). These also suggest that retroviruses can inhibit cGAS/STING signaling through multiple pathways.

#### 4.2.6 Others

The leucine-rich repeat-containing protein 10B(LRRC10B) up-regulated upon Sendai virus (SeV) infection participates in the formation and interaction with ankyrin repeat and SOCS box-containing 8 (ASB8)-TBK1/IKKϵ complex and negatively regulates IFN-I production by the degradation of TBK1 (Guo et al., 2020). In addition, ASB8 also interacts with PRRSV Nsp1α to promote K63-linked ubiquitination and increase the stability of Nsp1α, which facilitates the degradation of CBP to inhibit the production of IFN-I. Moreover, ASB8 also facilitates K48-linked ubiquitination and degradation of IKKβ, consequently suppressing NF-κB signaling (Han and Yoo, 2014) (Li et al., 2019). Thus, targeting ASB8 is a new strategy to enhance innate immunity. In addition, SeV infection promotes TBK1 s-nitrosation to suppress the innate immune response by inducing RNS (Liu et al., 2021). Of note, SeV can indirectly mediate IRF3 degradation by regulating the ubiquitination of IRF3. For example, SeV infection promotes 26S proteasome non-ATPase regulatory subunit 14 (PSMD14) to disassociate with IRF3. Then IRF3 is conjugated with K27-linked ubiquitin chains, which mediates the IRF3 autophagic degradation in a nuclear dot protein 52 (NDP52)-dependent manner (Wu et al., 2021). Furthermore, SeV infection promotes the nuclear translocation of TRIM26 and up-regulates the RBCK1 expression to degrade IRF3 (Zhang et al., 2008; Wang et al., 2015).

In addition, Guertu virus (GTV) is a potentially highly pathogenic bunyavirus. It has been reported that GTV nonstructural protein (NSs) induces the formation of compact inclusion bodies (IBs) and extended filamentous structures (FSs). Then, GTV NSs interacts with TBK1 and STAT2 to sequester them into the viral IBs and FSs, which counteracts the host’s innate immunity (Min et al., 2020). Furthermore, Heartland virus (HRTV) is a pathogenic phlebovirus, whose NSs protein inhibits innate immunity by interacting with TBK1 to hinder the association of TBK1 with IRF3 (Ning et al., 2017). HRTV also antagonizes IFN antiviral signaling by diminishing both STAT2 and STAT1 phosphorylation. Moreover, decreased STAT2 activity is involved in HRTV NSs (Feng et al., 2019). However, the underlying mechanism of reduced STAT1 phosphorylation remains unclear. TRIM6 can activate the IKKϵ kinase to induce IFN-I responses (Rajsbaum et al., 2014). However, it has been found that Nipah Virus matrix protein (NiV-M) interacts with TRIM6 and suppresses TRIM6-mediated IFN-I signaling (Bharaj et al., 2016). Also, Ebola virus (EBOV) VP35 interacts with host TRIM6 to inhibit the TRIM6-mediated IFN-I induction (Bharaj et al., 2017). Thus, the virus can escape immune response by hijacking the antiviral effect of TRIM6.

## 5 Summary

cGAS/STING signaling plays an essential role in the process of innate antiviral immunity. Not only DNA virus infection but also RNA virus infection can initiate the activation of cGAS/STING signaling. RNA viruses can activate cGAS/STING signaling by increasing mtDNA and chromatin DNA in the cytoplasm. Notably, activation of DNA sensors DNA-PK and AIM2 can also suppress the activation of cGAS, which may be a negative feedback regulation mechanism formed by the immune system to avoid the excessive activation of its innate immune signals and prevent the occurrence of autoimmune diseases. In addition, cGAS and its mediated downstream antiviral signals are regulated by numerous factors. Numerous studies have revealed that most viral proteins directly interfere with cGAS/STING signal transduction by directly targeting essential proteins such as cGAS, STING, and TBK1. However, viruses can also indirectly inhibit the activation of cGAS/STING signaling through some related regulatory factors. For example, viral infection can cleave K63-linked ubiquitin chains from STING by increasing MYSM1 levels or enhancing the recruitment of PPM1G to dephosphorylate STING. Viral infection can also reduce PTEN expression to inhibit nuclear translocation of IRF-3. However, whether the virus can inhibit cGAS/STING signaling through various other factors remains unclear. The modulation of cGAS/STING signaling has been applied not only to the treatment of DNA virus infections but also to RNA virus infections. However, current therapeutic research for viral infections has concentrated on the use of broad-spectrum therapeutic approaches that activate cGAS/STING signaling. For example, topoisomerase II inhibitors trigger the DNA-sensing cGAS-STING pathway that restricts EBOV replication (Luthra et al., 2017). The STING agonist 5,6-dimethylxanthenone-4-acetic acid (DMXAA) can inhibit HBV replication in a chronic HBV mouse model (Li et al., 2022). In addition, diABZI, the potent non-CDN STING agonist, can restrict SARS-CoV-2 replication in human bronchial epithelial cells and mice, in which the process is IFN-I signaling dependent (Li et al., 2021). These studies suggest that the strategy of employing STING agonists during viral infection can, to some extent, combat viral infection. Moreover, PLpro is a tempting target for broad-spectrum anti-coronavirus drugs. F0213, a broad-spectrum anti-coronavirus PLpro inhibitor, can interact with SARS2-PLpro and MERS-PLpro to antagonize PLpro-mediated IFN suppression. F0213 effectively protects hamsters infected with SARS-CoV-2 and human DPP4-knockin mice infected with MERS-CoV (Yuan et al., 2022). Thus, targeting critical factors according to the strategy of viral inhibition of cGAS/STING may be able to restore hijacked cGAS/STING signaling better than directly activating STING signaling with STING agonists. Therefore, targeting these factors to reactivate cGAS/STING signaling for specific viral infections is a novel viral therapy strategy.

## Author contributions

ZG is responsible for the retrieval of relevant documents and the writing of article content, and SD is responsible for the design of the overall framework of the article. All authors contributed to the article and approved the submitted version.

## Funding

The present study was supported by the National Natural Science Foundation of China (grant no. 31671241) and the Scientific Research Start-up Fund for Young Teachers of Shenzhen University.

## Conflict of interest

The authors declare that the research was conducted in the absence of any commercial or financial relationships that could be construed as a potential conflict of interest.

## Publisher’s note

All claims expressed in this article are solely those of the authors and do not necessarily represent those of their affiliated organizations, or those of the publisher, the editors and the reviewers. Any product that may be evaluated in this article, or claim that may be made by its manufacturer, is not guaranteed or endorsed by the publisher.
